# Clinical Utility of Liquid Comprehensive Genomic Profiling in Advanced Lung Adenocarcinoma Complicated by Disseminated Intravascular Coagulation: A Case Report

**DOI:** 10.7759/cureus.115692

**Published:** 2026-09-02

**Authors:** Hiromasa Sakurai, Hiroki Wakabayashi, Nanako Akiyama, Kenta Takashima, Kazutoshi Isobe

**Affiliations:** 1 Division of Respiratory Medicine, Department of Internal Medicine, Toho University Sakura Medical Center, Chiba, JPN

**Keywords:** adenocarcinoma, cancer genomic profiling, disseminated intravascular coagulation, her2 mutation, liquid biopsy, trastuzumab deruxtecan

## Abstract

Comprehensive genomic profiling (CGP) is increasingly important for identifying actionable driver mutations and selecting targeted therapies in advanced lung adenocarcinoma, particularly when tissue re-biopsy is precluded by severe complications. A 74-year-old woman with no history of smoking presented with hemoptysis and abnormal findings on chest imaging. She was diagnosed with lung adenocarcinoma, cT2bN2M1c, Stage IVB, with bone and brain metastases. Initial molecular testing using AmoyDx® (AmoyDx Essential NGS Panel, Amoy Diagnostics, Xiamen, China) did not reveal any actionable driver mutations. Despite multiple lines of systemic therapy and whole-brain irradiation, the disease progressed with multiple liver metastases and cancer-associated disseminated intravascular coagulation (DIC). Given the patient's never-smoking status and the lack of residual tissue for repeat testing, comprehensive liquid biopsy-based genomic profiling was performed using FoundationOne® Liquid CDx (Foundation Medicine, Boston, MA, US). This analysis identified a human epidermal growth factor receptor 2 (HER2) exon 20 insertion mutation (p.G776delinsAVGC). Treatment with trastuzumab deruxtecan (T-DXd) resulted in marked tumor shrinkage and prompt resolution of DIC. During the sixth cycle of T-DXd, the patient developed Grade 3 drug-induced interstitial lung disease (ILD)/pneumonitis, necessitating permanent discontinuation of T-DXd and high-dose corticosteroid therapy. Subsequently, the patient developed carcinomatous meningitis and was transitioned to palliative care. This case demonstrates that liquid biopsy-based CGP can identify actionable alterations and enable life-saving targeted therapy in patients with advanced lung adenocarcinoma complicated by severe DIC; however, severe drug-induced toxicities, such as ILD, remain a critical bottleneck that requires careful monitoring to balance treatment efficacy and safety.

## Introduction

In advanced lung adenocarcinoma, comprehensive genomic profiling (CGP) is increasingly important for identifying actionable driver alterations and selecting appropriate targeted therapies [1,2]. However, tissue-based CGP can be challenging or impossible in patients with severe, life-threatening complications such as cancer-associated disseminated intravascular coagulation (DIC). DIC is a severe clinicopathological syndrome characterized by systemic, uncontrolled activation of the coagulation cascade, leading to widespread microvascular thrombosis, secondary hyperfibrinolysis, and rapid consumption of platelets and clotting factors. Severe DIC results in profound consumption coagulopathy and marked thrombocytopenia, making invasive tissue biopsies, such as repeat lung or liver biopsies, clinically contraindicated due to an extraordinarily high risk of fatal bleeding [3].

Liquid biopsy-based CGP has emerged as a minimally invasive alternative that bypasses the need for tissue sampling. This approach analyzes circulating tumor DNA (ctDNA)-fragmented genomic DNA shed directly from primary and metastatic tumor cells into the circulating bloodstream. By isolating ctDNA from a routine blood draw, liquid biopsy enables comprehensive genomic evaluation without exposing high-risk patients to procedural bleeding hazards [1,2].

To appreciate the clinical utility of liquid biopsy in this setting, it is critical to distinguish between initial companion diagnostic testing and broad next-generation sequencing (NGS) [1,4]. Initial molecular screening frequently relies on multiplex polymerase chain reaction (PCR) assays, which utilize predefined primers targeting a narrow panel of common, high-frequency "hotspot" mutations [4]. In contrast, broad NGS platforms, such as FoundationOne® Liquid CDx (Foundation Medicine, Boston, MA, US), employ hybrid-capture technology to sequence hundreds of cancer-related genes simultaneously, detecting both common and rare alterations [2]. This technological distinction is particularly relevant for human epidermal growth factor receptor 2 (HER2) alterations. HER2 exon 20 insertions exhibit substantial molecular heterogeneity, encompassing diverse in-frame insertions and complex indels such as p.G776delinsAVGC. Because targeted PCR-based assays rely on specific primer designs, they often fail to detect rare or unconventional HER2 variants that fall outside their predefined target coverage [4]. Furthermore, single-site tissue biopsies are vulnerable to spatial and temporal intratumoral heterogeneity, leading to potential false-negative results [1,5]. Broad hybrid-capture NGS via liquid biopsy overcomes these limitations by analyzing ctDNA shed from multiple metastatic sites, capturing systemic tumor heterogeneity without the constraints of narrow PCR targeting [1,2].

HER2 mutations occur in 2%-4% of non-small cell lung cancer (NSCLC) [6,7], and targeting them has been revolutionized by trastuzumab deruxtecan (T-DXd)-an antibody-drug conjugate (ADC) designed to selectively deliver a potent topoisomerase I inhibitor payload to HER2-expressing tumor cells [8,9]. Here, we report a case of advanced lung adenocarcinoma in which an initial PCR-based assay failed to detect a driver mutation and DIC precluded repeat tissue biopsy. Liquid biopsy-based CGP using FoundationOne® Liquid CDx successfully identified a rare HER2 exon 20 insertion mutation (p.G776delinsAVGC), enabling targeted treatment with T-DXd that achieved rapid tumor regression and complete resolution of life-threatening DIC.

## Case presentation

A 74-year-old, never-smoking woman presented with hemoptysis and abnormal chest imaging findings. After a detailed evaluation, she was diagnosed with lung adenocarcinoma of the left lingular segment (cT2bN2M1c, stage IVB with bone and brain metastases; Figure 1), with a performance status of 0. Initial molecular testing using Amoy®Dx (AmoyDx Essential NGS Panel, Amoy Diagnostics, Xiamen, China) did not reveal detectable driver mutations; the programmed death-ligand 1 tumor proportion score was 1%-24%. 

**Figure 1:**
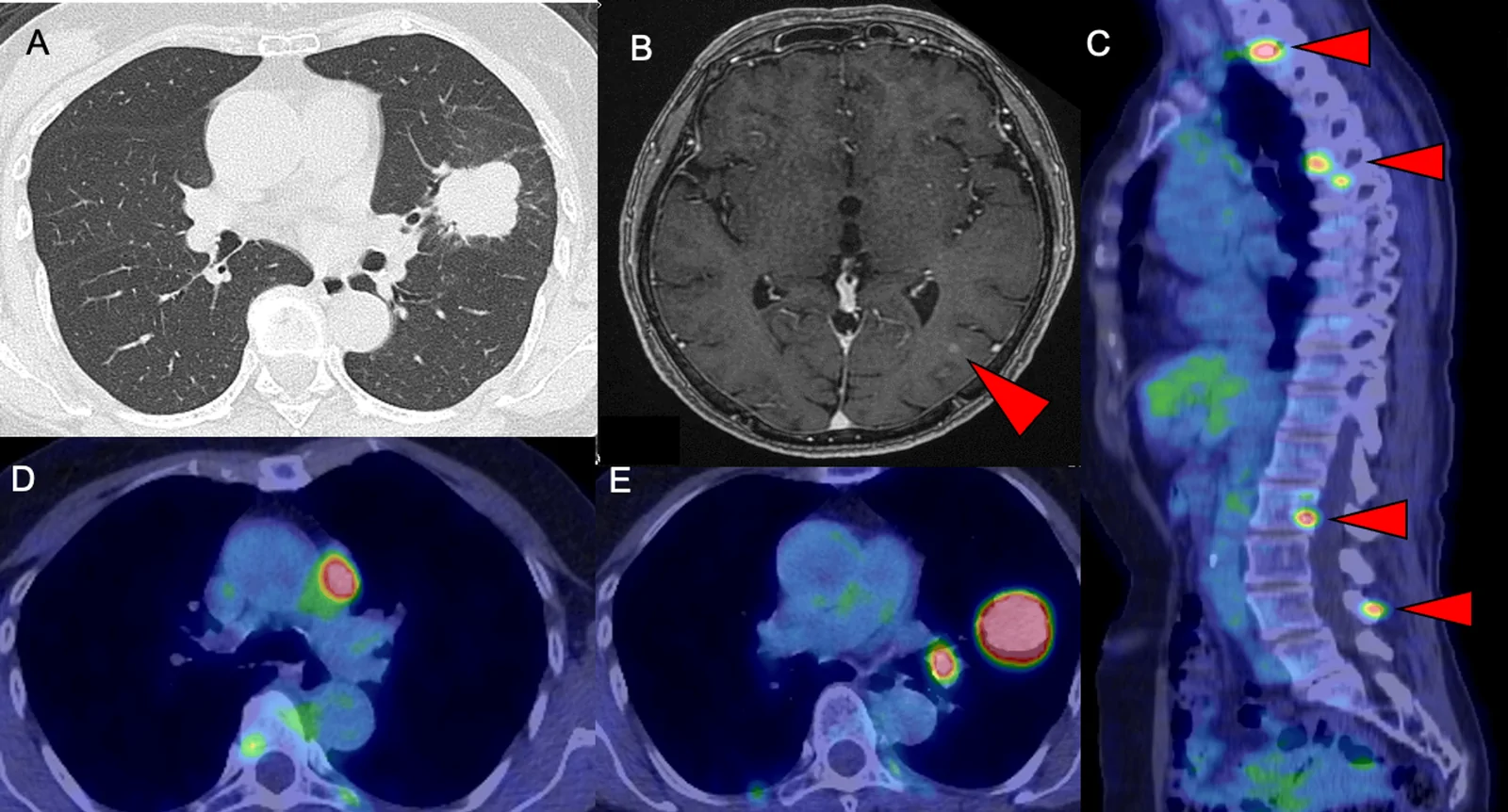
Imaging findings at the time of lung cancer diagnosis (A) Chest computed tomography scan showing a mass lesion in the left lingular segment. (B) Brain magnetic resonance imaging showing metastatic lesions in the brain (arrowhead). (C–E) Fluorodeoxyglucose (FDG) positron emission tomography showing increased FDG uptake in the primary lung tumor, hilar lymph node metastasis, and bone metastases (arrowheads).

The first-line treatment consisted of cisplatin plus pemetrexed and pembrolizumab for five cycles, and whole-brain radiotherapy was administered for brain metastases. This was followed by four cycles of docetaxel plus ramucirumab as second-line therapy, two cycles of S-1 as third-line therapy, and five cycles of atezolizumab, carboplatin, paclitaxel, and bevacizumab as fourth-line therapy. Despite these treatments, the primary tumor progressed, and multiple liver metastases developed (Figures 2A, 2C).

**Figure 2:**
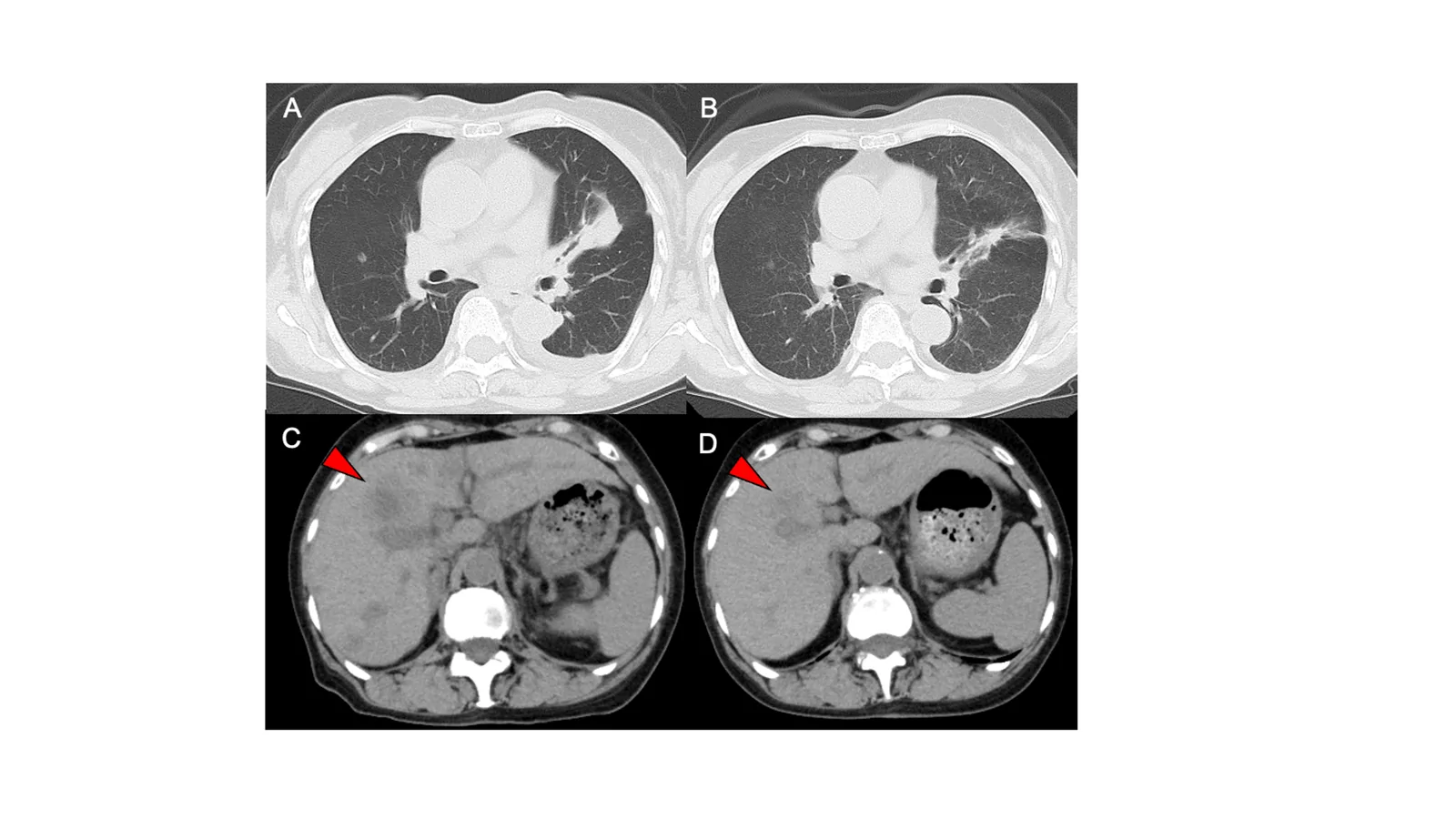
Radiological response to trastuzumab deruxtecan (T-DXd) (A) Chest computed tomography (CT) image obtained before T-DXd treatment. (B) Chest CT scan image after five cycles of T-DXd showing marked regression of the primary tumor. (C) Abdominal CT scan image before T-DXd treatment. (D) Abdominal CT scan image acquired after five cycles of T-DXd showing shrinkage of liver metastatic tumors (arrowhead).

Upon admission, the patient’s CRP level was elevated to 9.5 mg/dL. The platelet count decreased to 108,000/μL, while the D-dimer level increased to 54.65 μg/mL. According to the Japanese Association for Acute Medicine (JAAM) DIC criteria, the patient met the diagnostic threshold for DIC with a total score of 5 points (a score of ≥ 4 indicates DIC). Specifically, 1 point was scored for meeting the SIRS criteria (white blood cell count, 18,000/μL; threshold > 12,000/μL), 1 point for moderate thrombocytopenia (platelet count, 108,000/μL; threshold < 120,000/μL), and 3 points for marked elevation of D-dimer (54.65 μg/mL; threshold ≥ 25 μg/mL). The prothrombin time ratio remained within normal limits (PT-INR 0.96; threshold < 1.2) and scored 0 points [3]. Tumor markers showed a markedly elevated CEA level of 1561.2 ng/mL. The patient's laboratory findings on admission are summarized in Table 1.

**Table 1:** Laboratory values on admission ALP, alkaline phosphatase; ALT, alanine aminotransferase; APTT, activated partial thromboplastin time; AST, aspartate aminotransferase; BUN, blood urea nitrogen; CK, creatine kinase; CRP, C-reactive protein; LDH, lactate dehydrogenase; PT-INR, prothrombin time-international normalized ratio

Laboratory Parameter	Value	Unit
RBC	234	×10⁴/μL
Hb	10.2	g/dL
Platelet count	10.8	×10⁴/μL
WBC	18,000	/μL
Neutrophils	69.4	%
Lymphocytes	18.7	%
Monocytes	11.5	%
Eosinophils	0.2	%
Basophils	0.2	%
CRP	9.50	mg/dL
TP	6.7	g/dL
Albumin	2.9	g/dL
AST	100	IU/L
ALT	130	IU/L
LDH	394	U/L
ALP	318	U/L
γ-GTP	391	IU/L
Total bilirubin	0.6	mg/dL
CK	23	IU/L
BUN	13.2	mg/dL
Creatinine	0.59	mg/dL
Uric acid	3.6	mg/dL
Sodium	134	mEq/L
Potassium	3.9	mEq/L
Chloride	101	mEq/L
Calcium	8.8	mg/dL
Phosphorus	2.4	mg/dL
PT-INR	0.96	
APTT	22.2	S
D-dimer	54.65	μg/mL
CEA	1561.2	ng/mL
CA19-9	>70,000	U/mL
SLX	6760	U/mL

Since the patient was a never-smoker, we suspected the presence of an undetected oncogenic driver mutation. However, the tissue biopsy performed at the time of lung cancer diagnosis did not yield residual samples for CGP testing, and a repeat biopsy was deemed high-risk due to DIC. Therefore, we performed CGP based on liquid biopsy using FoundationOne® Liquid CDx, which identified a HER2 exon 20 insertion mutation (p.G776delinsAVGC).

Based on these findings, T-DXd was subsequently administered at a dose of 260 mg (5.4 mg/kg) every 3 weeks, together with continuous intravenous anticoagulation therapy using unfractionated heparin (15,000 U/day). Blood samples were collected daily. Serial assessments included platelet count, fibrin/fibrinogen degradation products, D-dimer, prothrombin time, fibrinogen level, clinical bleeding or thrombotic events, oxygenation status, performance status, and radiological tumor response. The heparin dose was adjusted to maintain the activated partial thromboplastin time at 1.5-2.5 times the control value. Following T-DXd administration, D-dimer levels decreased from 5.5 μg/mL on day 25 to 2.6 μg/mL on day 60, and the DIC score improved to 0. After five cycles of T-DXd, computed tomography (CT) demonstrated a reduction in the size of the primary tumor and liver metastases (Figures 2B, 2D), along with improvement of DIC (Figure 3).

**Figure 3:**
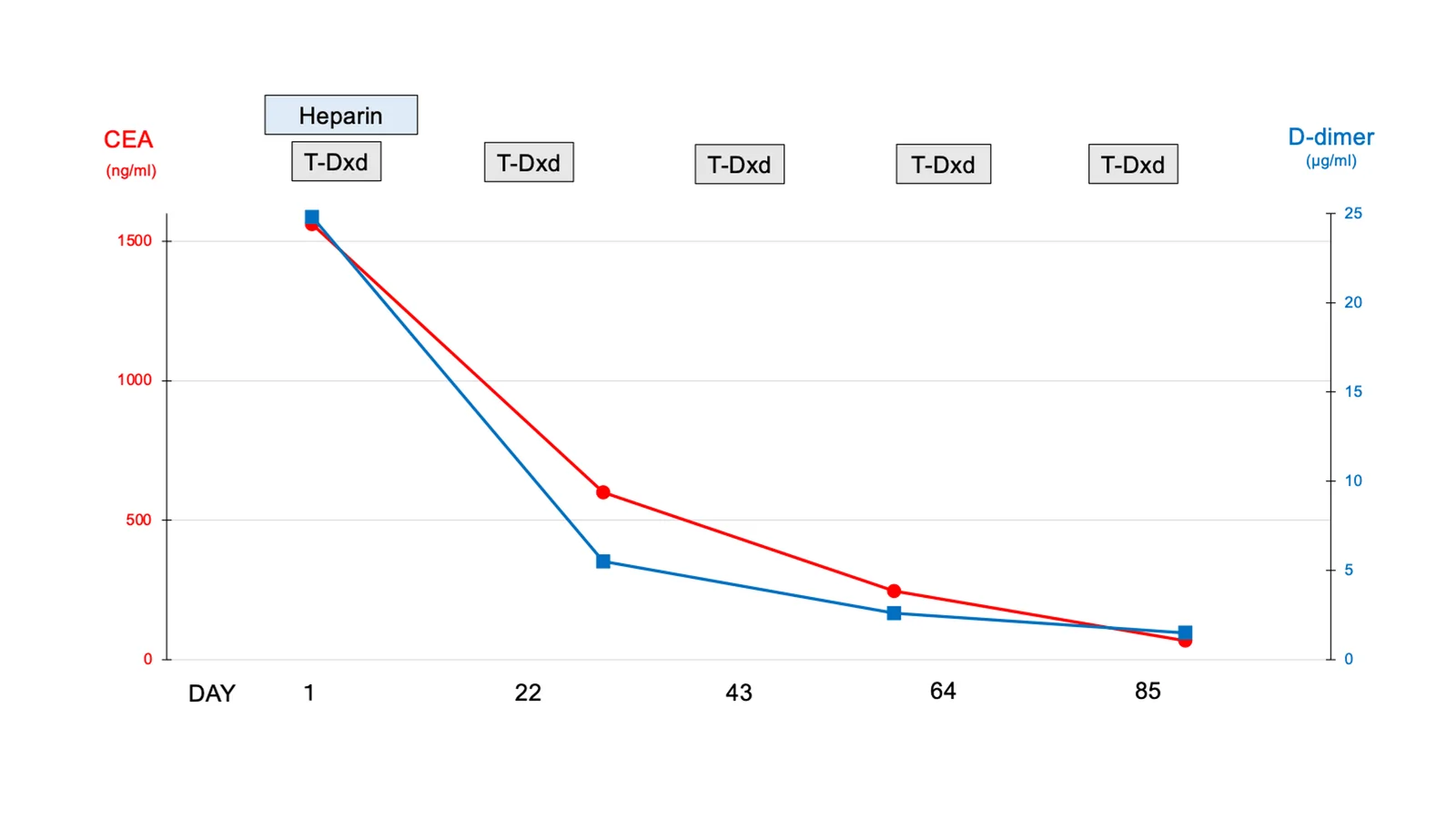
Clinical course The figure illustrates the clinical course and changes in serum CEA and D-dimer levels after initiation of T-DXd. T-DXd was administered at a dose of 260 mg every three weeks. Continuous intravenous heparin at 15,000 U/day was administered for DIC. Following the initiation of T-DXd, serum CEA and D-dimer levels progressively decreased, accompanied by improvement in DIC. CEA, carcinoembryonic antigen; DIC, disseminated intravascular coagulation; T-DXd, trastuzumab deruxtecan

During the sixth cycle of T-DXd treatment (about 17 weeks after starting), the patient suddenly developed a dry cough, worsening shortness of breath, and a low-grade fever. Blood tests showed an elevated KL-6 level (528 U/mL). Blood oxygen levels were low, with an oxygen saturation (SpO2) of 92% on room air. A chest CT scan showed new ground-glass opacities and patchy shadows in both lungs. Based on these findings, the patient was diagnosed with Grade 3 T-DXd-induced interstitial lung disease (ILD)/pneumonitis according to Common Terminology Criteria for Adverse Events (CTCAE) v5.0 [10]. T-DXd was stopped permanently, and high-dose systemic steroid treatment (prednisolone, 80 mg/day) was started immediately. After starting steroids, the patient's breathing symptoms, oxygen levels, and CT scan findings gradually improved.

## Discussion

In patients with advanced NSCLC, clinical findings might suggest the presence of a driver oncogene alteration. However, initial companion diagnostic testing may fail to detect actionable driver alterations [4]. Tissue-based CGP is generally performed using available tumor tissue when sufficient pathological material is available [5]. However, residual specimens obtained during the initial diagnostic procedure may be insufficient for CGP. Although a repeat biopsy can be considered, invasive tissue biopsy can be difficult or risky in patients with serious complications such as DIC, worsening general condition, or rapid disease progression. In this case, extensive liver metastases and tumor-associated DIC substantially increased the risk of bleeding, making repeat tissue biopsy difficult [3,5]. Accordingly, liquid biopsy-based CGP using FoundationOne® Liquid CDx was selected as a minimally invasive alternative [1,2]. This assay successfully identified a rare HER2 exon 20 insertion mutation (p.G776delinsAVGC), which had not been detected by the initial multiplex companion diagnostic assay. In the present case, the discrepancy between the initial negative result with AmoyDx Essential NGS Panel and the subsequent identification of the HER2 mutation by liquid biopsy can be attributed to differences in assay coverage and tissue sample conditions, rather than a simple superiority of NGS over multiplex PCR [4,6]. First, although newer multiplex PCR assays serve as companion diagnostics for HER2 alterations, targeted PCR panels rely on specific primer designs that typically cover only high-frequency hotspot variants such as A775_G776insYVMA. Unconventional or rare variants, such as the p.G776delinsAVGC insertion identified here, fall outside the predefined target coverage of such multiplex PCR assays [4,6]. Second, the limited volume of the initial tissue specimen, combined with potential spatial intratumoral heterogeneity, further increased the risk of a false-negative result on tissue testing [1,5]. Comprehensive hybrid-capture NGS via FoundationOne® Liquid CDx addresses these limitations by analyzing ctDNA derived from multiple metastatic sites without the constraints of narrow PCR primer targeting, thereby enabling the detection of rare, clinically actionable driver mutations [1,2,6,7].

In real-world thoracic oncology, clinicians frequently encounter scenarios where tissue or liquid biopsy alone is insufficient. Emerging evidence supports the clinical importance of combining tissue- and liquid-based genomic profiling (a complementary or concurrent testing strategy) [1,5]. Studies have demonstrated high concordance rates (often exceeding 80-90% for major oncogenic drivers) between tissue and blood NGS, particularly in patients with high systemic tumor burden and active DNA shedding [1,5]. However, discordance can occur due to spatial/temporal intratumoral heterogeneity, low plasma ctDNA shedding, or limited tissue availability [1,5]. While tissue biopsy remains the gold standard for histological diagnosis and PD-L1 evaluation, liquid biopsy provides a rapid, non-invasive overview of the systemic clonal architecture without procedural risks [1,5]. Utilizing both modalities concurrently or sequentially enhances driver detection rates, minimizes false negatives, and ensures that actionable alterations, such as rare HER2 insertions, are not missed in critically ill patients where diagnostic delays can be fatal [1,5].

Identifying this therapeutically targeted mutation enabled treatment with T-DXd [8,9]. Following T-DXd administration, the patient showed radiographic tumor reduction and DIC improvement [8,9]. These results suggest that liquid biopsy-based CGP may have significant therapeutic implications, even in severely ill patients with advanced cancer complicated by DIC and lacking residual samples at diagnosis [1,2].

HER2 mutations are identified in 2%-4% of lung adenocarcinomas, being more common in women, never-smokers, and patients with adenocarcinoma histology. HER2 exon 20 insertion mutations represent a heterogeneous molecular subgroup, and clinical data regarding rare variants, such as p.G776delinsAVGC, remain limited [6,7]. Nevertheless, accumulating evidence indicates that HER2-targeted therapies, including T-DXd, can significantly improve HER2-mutant NSCLC [8,9].

HER2 mutations in NSCLC represent a distinct molecular subclass that differs fundamentally from HER2 amplification or overexpression. Unlike EGFR exon 19 deletions or L858R mutations, HER2 exon 20 insertions are highly heterogeneous, comprising over 25 distinct in-frame insertion variants [6,7]. While the A775_G776insYVMA duplication accounts for ~80% of cases, uncommon variants, such as the p.G776delinsAVGC identified in our patient, present unique structural conformations that alter the drug-binding pocket [6,7]. Furthermore, HER2 mutations can present as uncommon clinical phenotypes or secondary resistance mechanisms. Secondary resistance to HER2-targeted agents, including T-DXd, can develop through diverse pathways, such as secondary HER2 kinase domain mutations (e.g., C805S, L726F), HER2 gene copy number alterations, or bypass pathway activation such as MET amplification, PIK3CA mutations, or histological transformation to small-cell lung cancer [6,8,9]. Recognizing these uncommon genomic presentations and resistance patterns underscores the necessity of broad hybrid-capture NGS, via both tissue and liquid platforms, to track tumor evolution and guide subsequent lines of targeted therapy [1,5,6].

Drug-induced ILD/pneumonitis is a well-known, potentially life-threatening adverse event associated with T-DXd therapy [8,9]. In pivotal clinical trials, such as DESTINY-Lung01 and DESTINY-Lung02, any-grade ILD was reported in 12-26% of patients with HER2-mutant NSCLC, with Grade 3 or higher events occurring in a subset of cases [8,9]. In the present case, treatment with T-DXd achieved rapid, clinically significant regression of liver metastases and prompt resolution of life-threatening DIC, leading to transient clinical stabilization. However, the patient subsequently developed Grade 3 ILD during the sixth cycle, necessitating permanent treatment discontinuation and transition to best supportive care.

This clinical trajectory highlights a critical clinical trade-off in the management of high-risk, advanced NSCLC complicated by severe DIC. On one hand, given the critical nature of DIC, swift initiation of highly effective targeted therapy, such as T-DXd, was essential for rapid tumor burden reduction and systemic disease control [8,9]. On the other hand, the emergence of severe pulmonary toxicity represents a major therapeutic bottleneck that can prematurely curtail an otherwise effective treatment. Close monitoring for early clinical symptoms, such as cough, dyspnea, and fever, and regular radiological surveillance are essential to balance efficacy and safety, enabling early intervention with high-dose corticosteroids before severe respiratory failure develops. Clinicians must carefully weigh the immediate benefits of rapid disease control against the inherent risks of severe ILD when employing T-DXd in fragile patients with oncological emergencies [8,9].

The patient's eventual progression to carcinomatous meningitis highlights the importance of understanding T-DXd's central nervous system (CNS) activity. Although ADCs are large molecules that typically have limited passive penetration through the blood-brain barrier (BBB), clinical evidence, including data from the DESTINY-Lung01 and DESTINY-Lung02 trials, shows that T-DXd has strong efficacy both systemically and intracranial in HER2-mutant NSCLC [8,9]. T-DXd carries a potent topoisomerase I inhibitor payload (DXd) with a high drug-to-antibody ratio (~8) [8,9]. Once released, the highly cell-permeable DXd payload exerts a strong bystander effect, allowing it to act effectively within the CNS tumor microenvironment where the BBB is locally disrupted [8,9].

In this case, T-DXd initially controlled the systemic metastases, but the onset of Grade 3 ILD required permanent drug discontinuation [8,9]. Pharmacologically, stopping T-DXd led to a loss of therapeutic drug levels in both systemic tissue and the CNS space. Without ongoing HER2-targeted inhibition, persistent micrometastases in the leptomeningeal space were able to grow rapidly, resulting in carcinomatous meningitis. This clinical course demonstrates that while T-DXd provides meaningful CNS activity, stopping treatment early due to severe adverse events can compromise intracranial control and lead to neurological relapse [8,9].

This case has two important clinical implications. First, a negative result on initial companion diagnostic tests does not necessarily rule out the presence of a treatable driver mutation. Second, CGP based on liquid biopsy may be particularly useful in patients for whom tissue biopsy is difficult, such as those without residual samples or with concomitant DIC.

While liquid biopsy-based CGP offers important clinical advantages, particularly for patients with critical complications, such as DIC, several technical limitations should be acknowledged. First, blood-based testing may yield false-negative results in the setting of low ctDNA shedding, low tumor burden, or localized disease [1]. Therefore, a negative liquid biopsy result does not exclude actionable driver alterations, and tissue-based testing remains the reference standard when clinically feasible [1,5]. Second, somatic variants detected in circulating cell-free DNA may occasionally arise from clonal hematopoiesis of indeterminate potential (CHIP) rather than from the tumor itself, although oncogenic driver alterations, such as HER2 exon 20 insertions, are rarely CHIP-derived [5]. Finally, variant allele fractions in liquid biopsy may fluctuate according to systemic tumor burden and ctDNA clearance [1,5]. Liquid biopsy results should therefore be interpreted in the context of the overall clinical, radiological, and pathological findings [1,5]. Repeat liquid biopsy or tissue re-biopsy should be considered when results are discordant, and the patient’s condition permits [1,5].

## Conclusions

Liquid biopsy-based CGP is a valuable diagnostic tool when tissue testing is precluded by limited sample volume or high bleeding risks, such as in cancer-associated DIC. In this case, identifying a rare HER2 exon 20 insertion (p.G776delinsAVGC) enabled targeted therapy with T-DXd, leading to rapid tumor regression and resolution of life-threatening DIC. However, the patient subsequently developed Grade 3 drug-induced ILD, requiring permanent drug discontinuation, which ultimately allowed rapid leptomeningeal progression and fatal neurological relapse. This clinical course illustrates the key trade-off of targeted therapy in fragile patients with oncological emergencies: while liquid biopsy can guide life-saving initial disease control, severe drug toxicities remain a major bottleneck that limits treatment durability and overall clinical benefit. Clinicians must balance prompt disease control with vigilant monitoring for treatment-limiting toxicities.
